# Real-world utilization patterns of systemic immunosuppressants among US adult patients with atopic dermatitis

**DOI:** 10.1371/journal.pone.0210517

**Published:** 2019-01-25

**Authors:** April W. Armstrong, Ahong Huang, Li Wang, Raymond Miao, Miraj Y. Patel, Abhijit Gadkari, Usha G. Mallya, Jingdong Chao

**Affiliations:** 1 Department of Dermatology, Keck School of Medicine at University of Southern California, Los Angeles, California, United States of America; 2 STATinMED Research/SIMR, Inc. Plano, Texas, United States of America; 3 Sanofi, Bridgewater, New Jersey, United States of America; 4 Regeneron Pharmaceuticals, Inc., Tarrytown, New York, United States of America; Charles P. Darby Children’s Research Institute, UNITED STATES

## Abstract

At the time of this study, prior to the introduction of biologics in the US, systemic therapies used for the treatment of moderate-to-severe atopic dermatitis included off-label immunosuppressants and corticosteroids. Immunosuppressant therapy is associated with a substantial risk of side-effects, therefore needing clinical monitoring, and is likely to incur a significant healthcare burden for patients and payers. This retrospective cohort study based on claims data measured immunosuppressant use and its associated burden among US adult patients with atopic dermatitis covered under commercial or Medicare Supplemental insurance from January 01, 2010, to September 30, 2015. Overall, based on age, gender, region, and index year, 4201 control patients with atopic dermatitis without immunosuppressant use were matched with 4204 patients treated with immunosuppressants. The majority (68.5%) of patients using immunosuppressants were non-persistent with immunosuppressant treatment during the 12-month follow-up period after a mean (standard deviation) of 88.1 (70.7) days of immunosuppressant use; 72.3% required systemic steroid rescue treatment. Immunosuppressant users had higher incidence of immunosuppressant-related clinical events than controls; in addition, a larger proportion of immunosuppressant users versus controls developed cancer (0.28% vs 0.14%, respectively; *P* < 0.0001). Healthcare utilization and costs associated with clinical events and monitoring were also higher for immunosuppressant users compared with controls (total costs, $9516 vs $1630, respectively; *P* < 0.0001; monitoring costs, $363 vs $54, respectively; *P* < 0.0001). This study revealed that patients treated with systemic immunosuppressants often require systemic steroids or changes to treatment. The increase in immunosuppressant-related clinical events, including the need for increased monitoring with immunosuppressant treatment, compared with controls demonstrates a substantial treatment burden and highlights the unmet need for more effective long-term therapies for atopic dermatitis with improved safety profiles and reduced monitoring requirements.

## Introduction

Atopic dermatitis (AD) is a chronic relapsing inflammatory skin disease that affects both children and adults [1, 2]. Symptoms include erythema, scaling and intense pruritus [1, 3]. AD can be associated with substantial disability and treatment burden, especially in patients with moderate-to-severe disease [4, 5]. This has a significant impact on society through increased healthcare resource utilization (HCRU) and costs, as well as on the individual through chronic sleep disruption, impaired health-related quality-of-life (HRQoL), and psychological problems [1, 6, 7].

Treatment of AD aims to reduce duration, severity and frequency of disease exacerbations (flares), and is implemented in a step-wise manner according to disease severity [8, 9]. Prior to the introduction of biologics, long-term systemic treatments were administered in moderate-to-severe AD patients uncontrolled on topicals; these generally included only off-label therapies such as immunosuppressants (IMMs) [3]. The literature suggests that the most common of these agents used in AD are cyclosporine, methotrexate, mycophenolate mofetil, and azathioprine [3, 10]. However, based on data in other disease areas, IMM treatment carries significant risk of side effects in addition to the increased risk of infection resulting from immunosuppression. In addition, toxicities can occur as a result of treatment with the IMMs most commonly used in AD; such toxicities might include nephrotoxicity (and associated contraindications in renal disease) with cyclosporine treatment, and hepatotoxicity with methotrexate [11] and azathioprine treatment [3, 12]. Furthermore, bone marrow suppression is likely to occur with treatment with methotrexate [11], azathioprine [12], and mycophenolate [13] leading to cytopenia. Risk of cutaneous and lymphoproliferative malignancies is also increased across all IMMs commonly used in AD [3]. This may also impact clinical considerations regarding phototherapy for AD due to the increased risk of these malignancies, and with methotrexate, increased photosensitivity is a further consideration [3, 11]. Monitoring requirements can also be considerable as a result of hematological, renal and/or hepatotoxic concerns with the IMMs commonly used in AD, with testing of certain clinical parameters (e.g. liver and renal function) recommended [3]. Side-effects such as gastrointestinal upset and flu-like symptoms [3] may further place a considerable burden on the patient. Furthermore, these treatments may be inappropriate in some patients due to drug-drug interactions, drug sensitivities (a possible side effect of all the IMMs most commonly used in AD), and contraindications [3]. As such, IMM treatment in patients with AD must be carefully based on clinical consideration of the individual patient.

The data regarding the limitations of IMMs are generally derived from patients with diseases other than AD. As IMMs are used off-label in treating AD in the US, evidence supporting their use is inconsistent and based on only a limited number of randomized controlled studies [3]. Guideline recommendations for the use of IMMs in treating AD are mostly consensus and expert opinion-based [3]. Definitions of patient eligibility for IMM treatment vary due to the subjective assessment of side-effects and additional treatment burden [3, 10]. All these issues lead to a lack of clarity on the appropriate use of IMMs in clinical practice [3, 10].

At present, there is a lack of understanding of real-world utilization patterns of IMMs and the associated consequences among adult patients with moderate-to-severe AD, highlighting the need for additional real-world evidence. By identifying IMM utilization patterns and the associated clinical monitoring in practice among patients with moderate-to-severe AD, the aim is to better understand the clinical and economic burden associated with their use. This in turn will reveal the potential need for safer and more efficacious therapies.

This retrospective cohort study presents real-world evidence on IMM utilization patterns among US adult AD patients, and the associated burden, including untoward clinical events, based on data from the Truven Health MarketScan database.

## Methods

### Study design

**Fig 1:**
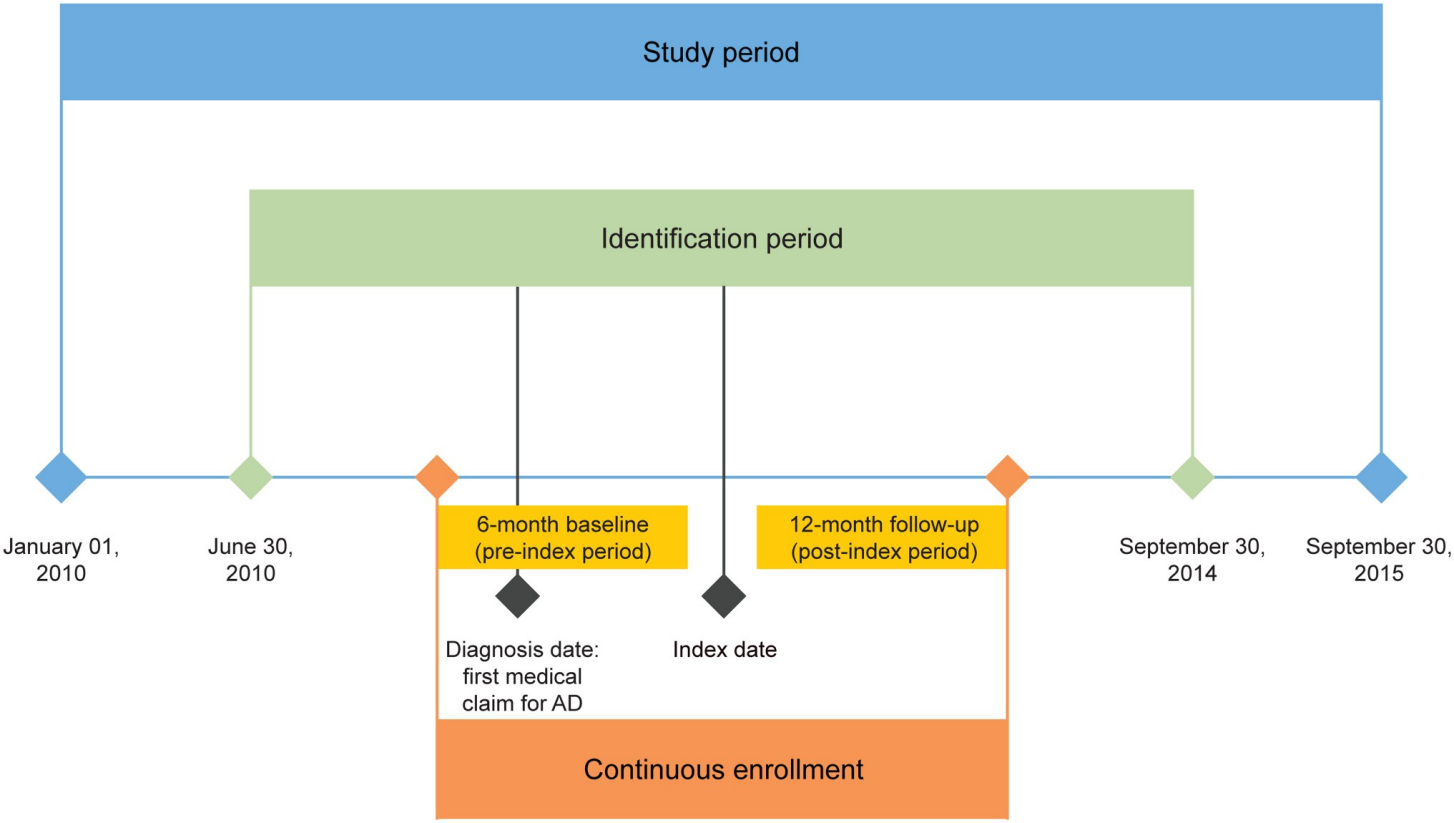
Study design. AD, atopic dermatitis.

This retrospective cohort study used claims data from the Truven Health MarketScan database from over 245 million anonymized patients [14]. Patients diagnosed with AD with commercial or Medicare Supplemental Insurance between January 01, 2010 and September 30, 2015 were identified in the database. The earliest physician diagnosis of AD in this period was designated as the initial AD diagnosis. Patients were included in the IMM group if they had a medical or pharmacy claim for a systemic IMM (cyclosporine, azathioprine, methotrexate, mycophenolate mofetil) between June 30, 2010, and September 30, 2014; the first such claim after the initial AD diagnosis was designated as the index date for this case group. AD patients without a claim for a systemic IMM, systemic corticosteroids (SCS), or phototherapy during the study period were categorized as controls; their index date was randomly assigned to a date within this period. For both case and control groups, the pre-index (baseline) period was 6 months prior to the index date. The post-index (follow-up) period was defined as a 12-month period on or after the index date. The study design is summarized in Fig 1.

### Inclusion and exclusion criteria

Patients ≥18 years with continuous health plan enrollment through the pre- and post-index period were included in the study if they met one of the following two criteria: a record(s) with a diagnosis code for AD (ICD-9-CM 691.8) during the study period; or a record(s) of a diagnosis for contact dermatitis due to an unspecified condition (ICD-9-CM 692.9) or rash and other non-specific skin eruption (782.1) during the identification period AND ≥2 diagnosis codes for asthma (493.xx), food allergies (V15.01–05), or allergic rhinitis (477.xx) over the study period.

Patients identified with evidence of other immunological diseases (such as rheumatoid arthritis, psoriatic arthritis, psoriasis, Crohn’s disease, ulcerative colitis, ankylosing spondylitis, idiopathic and non-infectious uveitis, or systemic lupus erythematosus), those who had undergone organ transplant, or those who had any IMM use during the baseline period were excluded.

Control patients were matched to systemic IMM patients on a 1:1 ratio based on demographics (birth year, gender, geographic region, and index year). If more than one matched control patient was available, only one would be randomly selected.

### Baseline variables

Socio-demographic variables captured at the index date included: age, gender, and US geographic region in accordance with the US Census Bureau designations. The Charlson Comorbidity Index (CCI) Score [15] (calculated using the Devo-modified protocol) [16, 17] was measured at baseline.

### Outcome variables

Clinical event rates related to individual IMM treatments were calculated for the IMMs included in the study. Only incident events during follow-up were measured. Treatment-related clinical events were identified using ICD-9-CM codes for clinical events potentially related to IMM treatment among AD patients and defined as ≥1 inpatient or ≥2 outpatient claims with IMM drug use within 90 days prior to the event’s diagnosis, or 12 months for any malignancies. This included: muscle cramps, gingival and periodontal diseases, acute kidney injury, infections (bacterial, viral, and fungal), hepatotoxicity, bone marrow suppression, lymphomas, skin cancers, any cancer, fever, myalgia, pulmonary fibrosis, abdominal pain, adverse drug events (for adverse drug reactions), bone loss, cataract, Cushing’s disease, diarrhea, erythema multiform of Erythema nodosum, glaucoma, glucose intolerance, headache, hypertrichosis, loss of appetite, myopathy, nausea, nausea and vomiting, nephrotoxicity, pneumonitis, urticaria, vasculitis, and vomiting. The 90 days of latency-time window was defined arbitrarily based on clinical judgement. Two different time windows of 30 and 60 days were also explored in the sensitivity analysis. For malignancy detection, a 12-month time window was used based on prior research.

The number of patients with IMM-related monitoring during the follow-up period was measured across a range of biomedical tests commonly carried out in patients treated with IMMs based on the presence of corresponding Healthcare Common Procedure Coding System codes. In addition, the HCRU and costs of patients with one or more IMM-related clinical event during the follow-up period were measured through the ICD-9 diagnosis codes for the clinical events.

The associated HCRU and costs for follow-up clinical event categories included: inpatient admissions, outpatient office or other visits, emergency room (ER) visits, and total medical costs (inpatient + outpatient). For inpatient admission, the length of stay (LOS) was also measured. Costs identified during the follow-up period were adjusted to 2015 US dollars using the annual medical care component and drugs cost component of the Consumer Price Index. Inpatient, ER, office and other costs, and total medical costs (outpatient + inpatient costs) were calculated.

Non-persistence of IMMs was defined as a gap of ≥90 days between the run-out date (date of prescription fill + days of supply) of an IMM and any subsequent prescription for IMM treatment.

Treatment outcomes during the 12-month follow up period included: concomitant treatment with oral/injection steroids, phototherapy, topical calcineurin inhibitor (TCI), or topical corticosteroid (TCS); switching from, or dose escalation of existing IMMs; use of additional IMMs; or hospitalization for IMM-related clinical events (a primary or secondary diagnosis of any IMM-related clinical events within 90 days of IMM fill, or 12 months for any malignancy).

### Data analysis

#### Statistical analysis

Frequencies were provided for dichotomous and polychotomous variables, with *P*-values calculated using the chi-square test for categorical variables and *t*-tests for continuous variables. Additionally, standardized differences (STDs) were also provided.

#### Generalized linear model

HCRU associated with IMM monitoring was compared between the IMM and control groups using a generalized linear model (GLM). Rates of IMM-related clinical events, monitoring events, and any HCRU were modeled using conditional logistic regression. The dependent variables were rate of events and patients with healthcare resource use; the independent variables included patient demographic data (age, gender, and region) and CCI scores. Costs were estimated based on the GLM and log transformation model.

## Results

### Demographics

Of 3,351,998 patients with an AD diagnosis initially identified in the database, 4204 IMM users were included in the analysis, matched with 4201 controls (three IMM users could not be matched with controls) based on birth year, gender, geographic region, and index year. Baseline characteristics are shown in Table 1. The most commonly used IMM was methotrexate, used by 51.3%, while 17.3%, 16.9%, and 14.5% used mycophenolate mofetil, cyclosporine, and azathioprine, respectively.

**Table 1:** Baseline patient characteristics.

	Azathioprine (n = 611)	Cyclosporine (n = 712)	Methotrexate (n = 2055)	Mycophenolate mofetil (n = 726)	IMM group (n = 4204)	Control group (n = 4201)^†^
Age, mean (SD) years	51.5 (15.1)	47.4 (16.0)***	51.5 (15.0)	51.9 (16.0)	50.9 (15.5)	50.9 (15.4)
Gender, n (%)						
Female	403 (66.0)	433 (60.8)**	1590 (73.8)***	410 (56.5)***	2836 (67.5)	2835 (67.5)
Male	208 (34.0)	279 (39.2)**	565 (26.2)***	316 (43.5)***	1368 (32.5)	1366 (32.5)
US region, n (%)						
Northeast	60 (9.8)**	134 (18.8)*	322 (14.9)	139 (19.2)*	655 (15.6)	655 (15.6)
North Central	137 (22.4)	107 (15.0)**	498 (23.1)	156 (21.5)	898 (21.4)	898 (21.4)
South	271 (44.4)	282 (39.6)	894 (41.5)	270 (37.2)	1717 (40.8)	1717 (40.9)
West	116 (19.0)	177 (24.9)*	386 (17.9)	142 (19.6)	821 (19.5)	820 (19.5)
Other	27 (4.4)*	12 (1.7)	55 (2.6)	19 (2.6)	113 (2.7)	111 (2.6)
Clinical characteristics						
Deyo-modified CCI score, mean (SD)	1.0 (1.5)***	0.6 (1.1)***	0.8 (1.4)***	1.2 (1.7)***	0.9 (1.4)***	0.4 (0.9)
CCI score = 0, n (%)	295 (48.3)***	457 (64.2)***	1277 (59.3)***	345 (47.5)***	2374 (56.5)***	3224 (76.7)
CCI score = 1, n (%)	189 (30.9)***	169 (23.7)***	491 (22.8)***	181 (24.9)***	1030 (24.5)***	630 (15.0)
CCI score = 2, n (%)	48 (7.9)**	44 (6.2)	199 (9.2)***	79 (10.9)***	370 (8.8)***	192 (4.6)
CCI score = 3, n (%)	43 (7.0)***	24 (3.4)*	106 (4.9)***	58 (8.0)***	231 (5.5)***	88 (2.1)
CCI score ≥ 4, n (%)	36 (5.9)***	18 (2.5)	82 (3.8)***	63 (8.7)***	199 (4.7)***	67 (1.6)

*P < 0.05;

**P < 0.001,

***P < 0.0001 (vs control group).

^†^Three IMM users could not be matched with controls.

CCI, Charlson Comorbidity Index; IMM, immunosuppressants; SD, standard deviation.

### IMM utilization patterns

**Fig 2:**
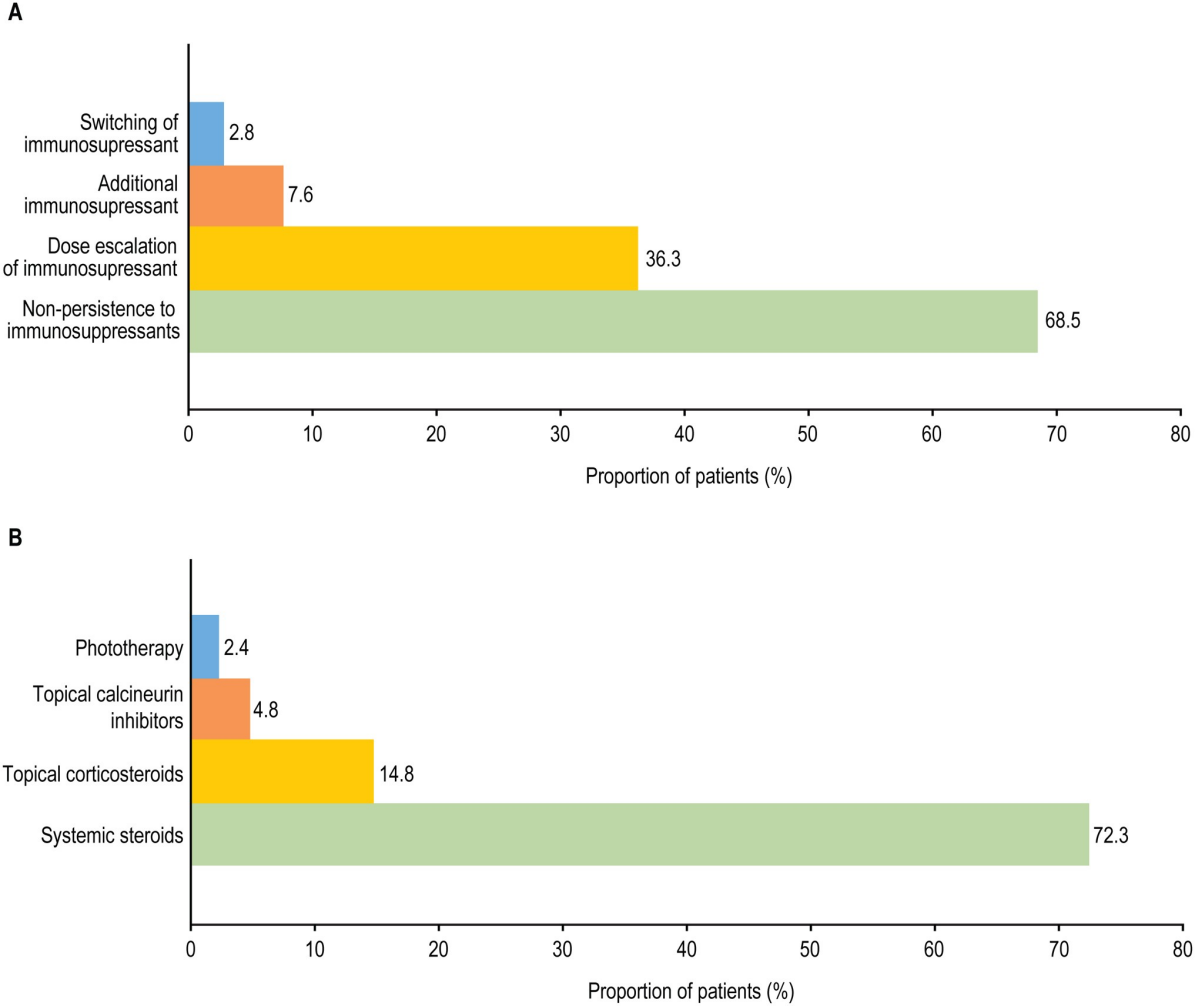
Systemic immunosuppressant treatment changes (n = 4204) (A) during 12-month follow-up period (B) and use of alternative therapies.

In the 12-month follow-up period, 2878 patients (68.5%) in the IMM group were non-persistent, while only 12.0% resumed IMM therapy after the treatment gap (Fig 2A); (**Table A in** S1 File). Mean (SD) time to IMM treatment gap date from index date was 88.1 (70.7) days. Overall, 3557 (84.6%) patients in the IMM group received concomitant treatment, which included oral corticosteroids or SCS (72.3%), TCS (14.8%), TCI (4.8%), and phototherapy (2.4%) (Fig 2B) (**Table A in** S1 File). Dose escalation was required for 36.3% of patients receiving IMM therapy, with 32.9% seeing an escalation in dose >10%. Additional IMMs were required for 7.6% of patients in the IMM group, and 2.8% of patients switched IMMs. The mean (SD) number of claims for systemic steroids was 4.6 (3.9).

### GLM-adjusted incidence of IMM-related clinical events

**Fig 3:**
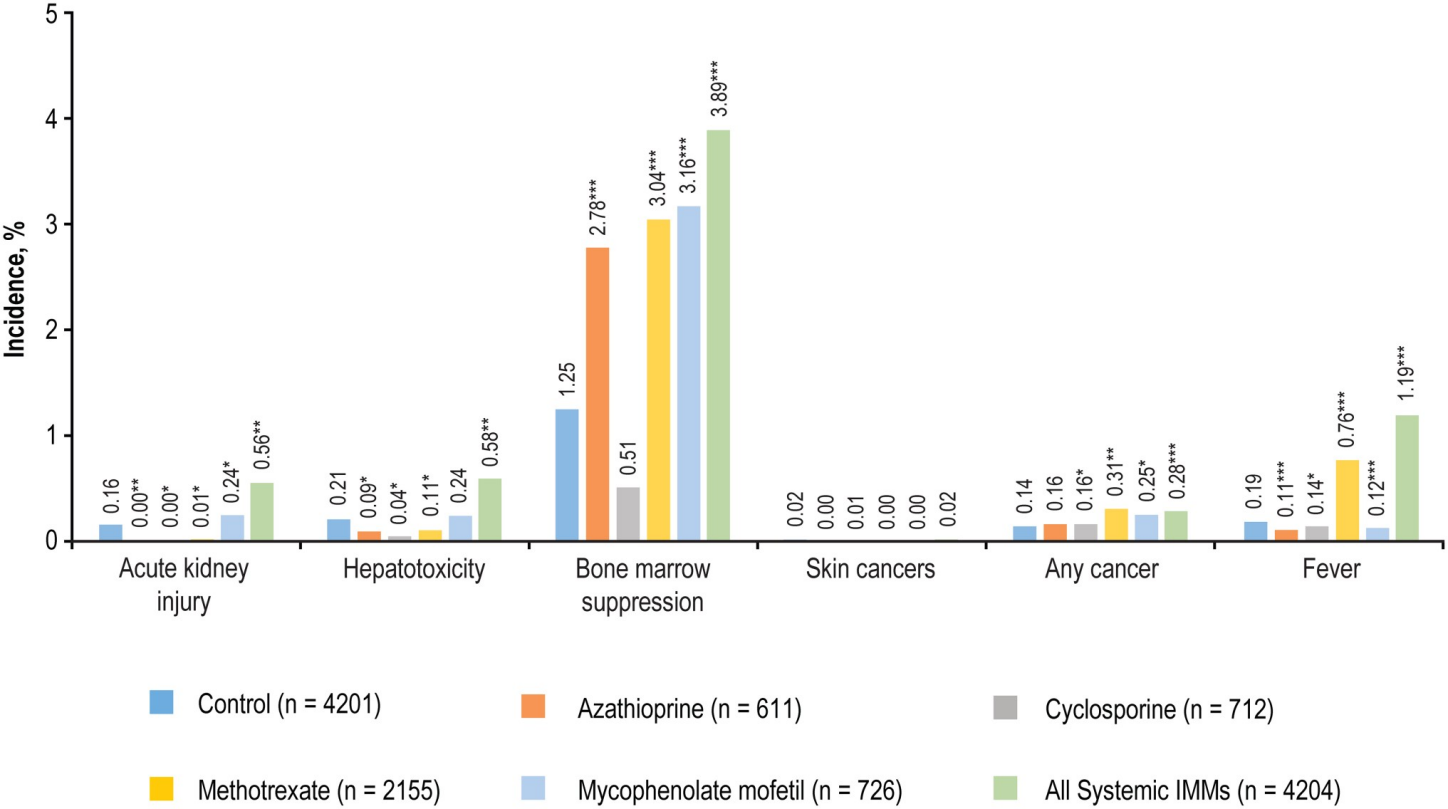
GLM-adjusted incidence of IMM-related clinical events in patients using systemic immunosuppressants vs controls. *P < 0.05; **P < 0.001; ***P < 0.0001. Controls shown represent the aggregated controls across all systemic IMMs. *P*-values of individual IMMs are in comparison with matched controls.

Compared with the control group, there were significantly higher rates of IMM-related clinical events in the IMM group (Fig 3). Significantly more patients in the IMM group were diagnosed with cancer compared with the control group, 0.28% vs 0.14%, *P* < 0.0001 (Fig 3) (**Table A in** S1 File).

The incidence of IMM-related clinical events resulting in hospitalization was significantly higher for the IMM group than for the control group (5.7% vs 2.0%, *P* < 0.0001), and for each individual IMM compared with the control group—azathioprine, 4.2 times higher; cyclosporine, 2.4 times higher; methotrexate, 2.5 times higher; mycophenolate, 3.8 times higher; all *P* < 0.0001. (**Table B in** S1 File).

Sensitivity analysis of prescription fills within 30 and 60 days prior to the clinical event diagnosis demonstrated a similar incidence rate of IMM-related clinical events compared with the 90-day period, with analysis across all periods suggesting significantly higher rates in the IMM group compared with the control group.

### GLM-adjusted healthcare resource utilization and costs associated with IMM-related clinical events

Across all categories assessed, patients in the IMM group had a higher level of HCRU and associated costs due to IMM-related clinical events compared with the control group (Table 2), including 5.6 times greater inpatient LOS, 4.5 times more inpatient visits, 3.0 times more outpatient ER visits, 2.6 times more outpatient visits, and 2.7 times more outpatient visits, all *P* < 0.0001.

**Table 2:** Generalized linear model-adjusted healthcare resource utilization and costs associated with IMM-related clinical events.

	IMMs (n = 4204)^†^	Control (n = 4201)	*P*-value
**Annual mean IMM-related clinical event-related HCRU per patient**			
Inpatient visits, %	6.9	2.2	< 0.0001
Outpatient ER visits, %	7.5	3.0	< 0.0001
Outpatient office visits, %	45.3	27.9	< 0.0001
Other outpatient visits, %	46.5	29.3	< 0.0001
Inpatient length of stay, days	0.45	0.08	< 0.0001
Number of inpatient visits, n	0.09	0.02	< 0.0001
Number of outpatient ER visits, n	0.12	0.04	< 0.0001
Number of outpatient office visits, n	1.75	0.68	< 0.0001
Number of other outpatient visits, n	2.25	0.84	< 0.0001
**Annual mean IMM-related clinical event-related healthcare costs per patient (US$)**			
Inpatient	4527	804	< 0.0001
Outpatient ER	125	43	< 0.0001
Outpatient office	332	98	< 0.0001
Other outpatient	4131	597	< 0.0001
Total medical (inpatient + outpatient)	9516	1630	< 0.0001

^†^Three IMM users could not be matched with controls.

ER, emergency room; HCRU, healthcare resource utilization.

### GLM-adjusted use of monitoring tests and associated costs

The proportion of patients who received at least one IMM-related monitoring test was significantly higher in the IMM group than in the control group (87.7% vs 61.4%; *P* < 0.0001), with the difference more pronounced for patients requiring at least two treatment-related monitoring tests (76.0% vs 27.1%, respectively; *P* < 0.0001). IMM-monitoring related costs per patient were significantly higher in the IMM group than in the control group (total costs, $363 vs $54; *P* < 0.0001).

## Discussion

Off-label use of IMMs for the treatment of AD has been recognized to have some effect in patients whose condition is uncontrolled with topical treatments [3]. However, there is less known about the wider consequences of their use. Hence, large-scale, real-word studies, not only measuring treatment utilization in routine clinical practice, but also the associated healthcare and economic burden in the US is highly warranted.

Our findings demonstrated that a large proportion of patients (68.5%) treated with systemic IMMs were non-persistent during the 12-month follow-up period, with only 12.0% subsequently resuming IMM treatment. These results from US patients are in line with previously reported real-world evidence from other countries, which demonstrated that systemic IMM treatment discontinuation was mostly related to adverse events and lack of efficacy [18]. In addition to a high non-persistence rate with systemic IMM treatment, our results demonstrate the need for other treatment changes such as dose escalation (36.3%), switching IMM treatment (2.8%), or adding another IMM treatment (7.6%). Additionally, patients commonly (72.3% of IMM patients) required multiple courses of rescue therapy with SCSs. This is in line with the current treatment guidelines for the management of AD, which recommend systemic steroids only for the treatment of acute, severe exacerbations, as transitional therapy to other systemic therapies in severe, rapidly progressive, or debilitating cases or during treatment optimization [3, 19]. These results, combined with the high non-persistence rate for systemic IMMs, suggest that IMMs provide inadequate control of AD symptoms.

Moreover, systemic IMM therapy is known to be associated with side effects ranging from headache and gastrointestinal side effects, to an increased risk of infection and certain cancers [3]. The clinical event profiles of systemic IMMs are well documented in respective product labels [11, 12, 20, 21]. For example, in the FDA label for cyclosporine [20], it is reported that 15.7 to 19.8% of psoriasis patients in clinical trials had increased levels of creatinine, with one case of death due to progressive renal failure. This is consistent with our results which demonstrated an increased risk of IMM-related clinical events in patients treated with systemic IMMs compared with those not treated with IMMs.

Not surprisingly, the occurrence of IMM-related clinical events during systemic IMM therapy was associated with significantly increased HCRU and costs across all categories assessed (both inpatient and outpatient visits, and inpatient LOS). The increased requirement for monitoring in patients using systemic IMM therapy was also resource-intensive and costly.

The strength of this study lies in its use of a large data sample of US patients with employer-provided health insurance, which may reflect the experience with systemic IMMs nationally.

A limitation of this study is that the presence of a diagnosis code on a medical claim does not necessarily indicate a positive presence of disease, due to being incorrectly coded or included as rule-out criteria rather than an actual disease. However, the use of AD-related medication might enhance the chance identification of AD, thus reducing the risk of misclassification. In addition, our study demonstrated a potential association between systemic IMM use and treatment burden and outcomes; however, since this is a retrospective database it is not possible to establish a direct causal relationship.

## Conclusions

To the best of our knowledge, this is the first real-world study on treatment patterns, adverse clinical event-related and monitoring-related HCRU and costs associated with systemic IMM treatment in AD patients. Our results demonstrate that IMM treatment in moderate-to-severe AD often provides unsatisfactory outcomes, with most (72.3%) moderate-to-severe AD patients treated with IMMs requiring systemic steroid rescue therapy. In addition, more than two-thirds of patients are non-persistent with IMMs. Finally, IMMs are associated with adverse clinical events, with 75% of users requiring at least one monitoring test during the 12-month follow-up period. These results highlight the unmet need for more targeted, safe, and effective long-term treatment options for patients with moderate-to-severe AD.

## Supporting information

S1 File Treatment outcomes and resource use of atopic dermatitis treated with immunomodulating agents. Table A in S1 File. Treatment Outcomes Among AD Patients. Table B in S1 File. GLM-Adjusted Hospitalization for IMM-related clinical events.
(XLSX)
